# Mast cells in the bone marrow microenvironment

**DOI:** 10.3389/fimmu.2025.1738487

**Published:** 2026-01-02

**Authors:** Domenico Ribatti

**Affiliations:** Department of Translational Biomedicine and Neuroscience, University of Bari, Bari, Italy

**Keywords:** angiogenesis, bone marrow, fibrosis, mast cells, tumor progression

## Abstract

The bone marrow microenvironment provides the necessary signals for the development from hematopoietic stem cells into mast cell precursors. Once released in the bloodstream, mast cells quickly migrate to peripheral tissues where they complete their differentiation. Mast cells are located at the interface with the external environment, therefore are among the first cell types that can get in contact with pathogens. Mast cells are found in every tissue, most abundantly near barriers where they exert the role of immune sentinels during both acute and chronic inflammation. Mast cells are known for their effects when eliciting immediate hypersensitivity reactions. However, they are involved in numerous other physiological and pathological conditions such as those that occur in bone marrow microenvironment, as has been discussed in this article.

## Mast cells in the bone marrow microenvironment in physiological conditions

Bone marrow microenvironment is composed of hematopoietic stem cells (HSCs) and nonhematopoietic cells, including endothelial cells, pericytes, endothelial progenitor cells, fibroblasts, osteoblasts, osteoclasts, mast cells, macrophages, and mesenchymal stem cells, that provide structural support and are involved in normal hematopoiesis (1).

**Figure 1:**
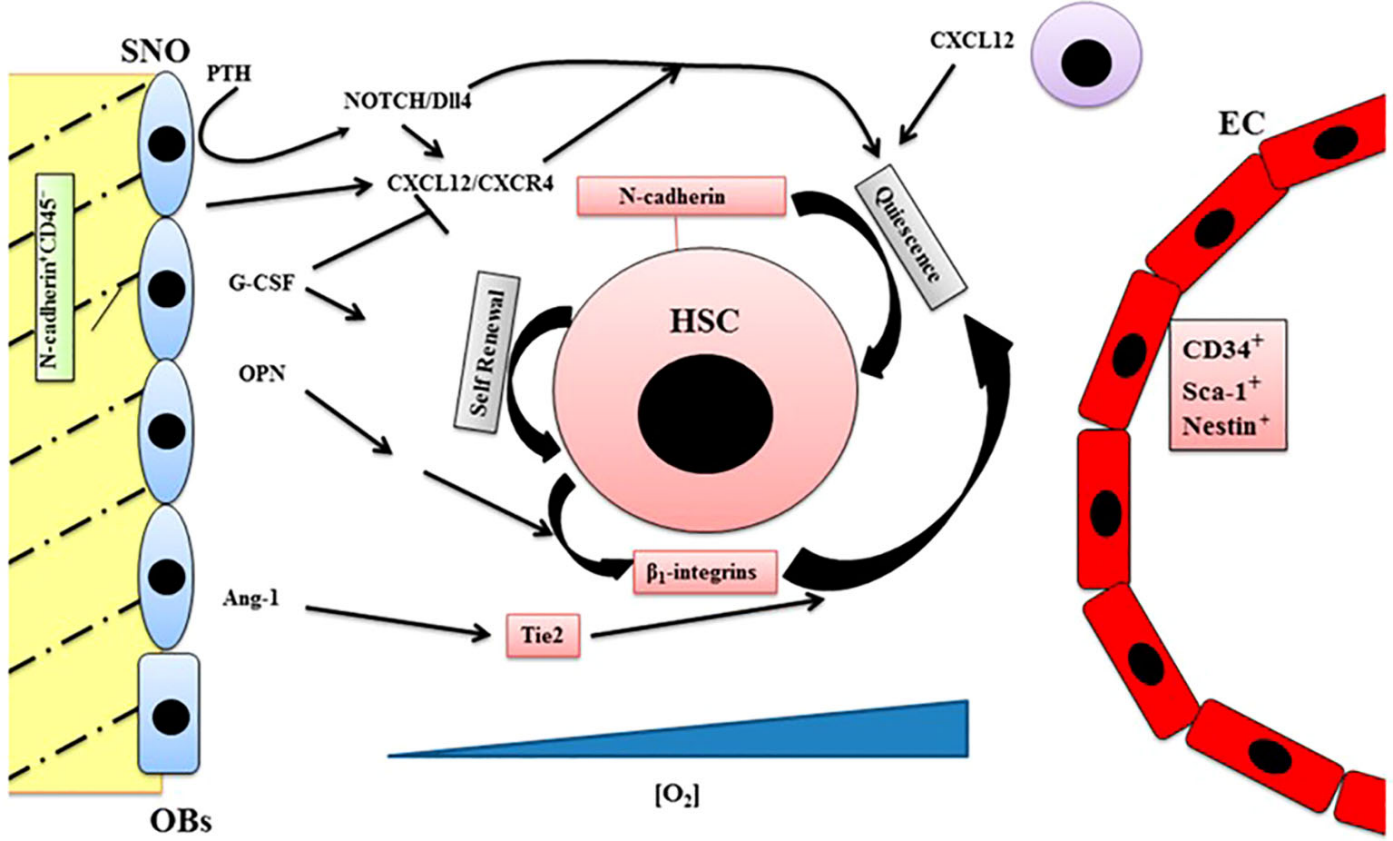
The endosteal niche is a complex structure inside which all the components, such as stem cells, progenitor cells, stromal cells, growth factors, and extracellular matrix (ECM) molecules participate in the regulation of hematopoiesis. Spindle-shaped N-cadherin^+^CD45^−^ osteoblastic cells (SNO); osteoblasts (OBs); endothelial cells (EC); hematopoietic stem cells (HSC); granulocyte colony-stimulating factor (G-CSF); osteopontin (OPN); parathyroid hormone (PTH); δ like ligand 4 (Dll4); C-X-C chemokine receptor type 4 (CXCR4); stromal cell derived factor-1/C-X-C motif chemokine 12 (CXCL12); angiopoietin receptor-2 (Tie2); angiopoietin-1 (Ang-1); Notch/translocation−associated Notch homologue (NOTCH). Blue triangle: oxygen gradient. (Reproduced from 2).

**Figure 2:**
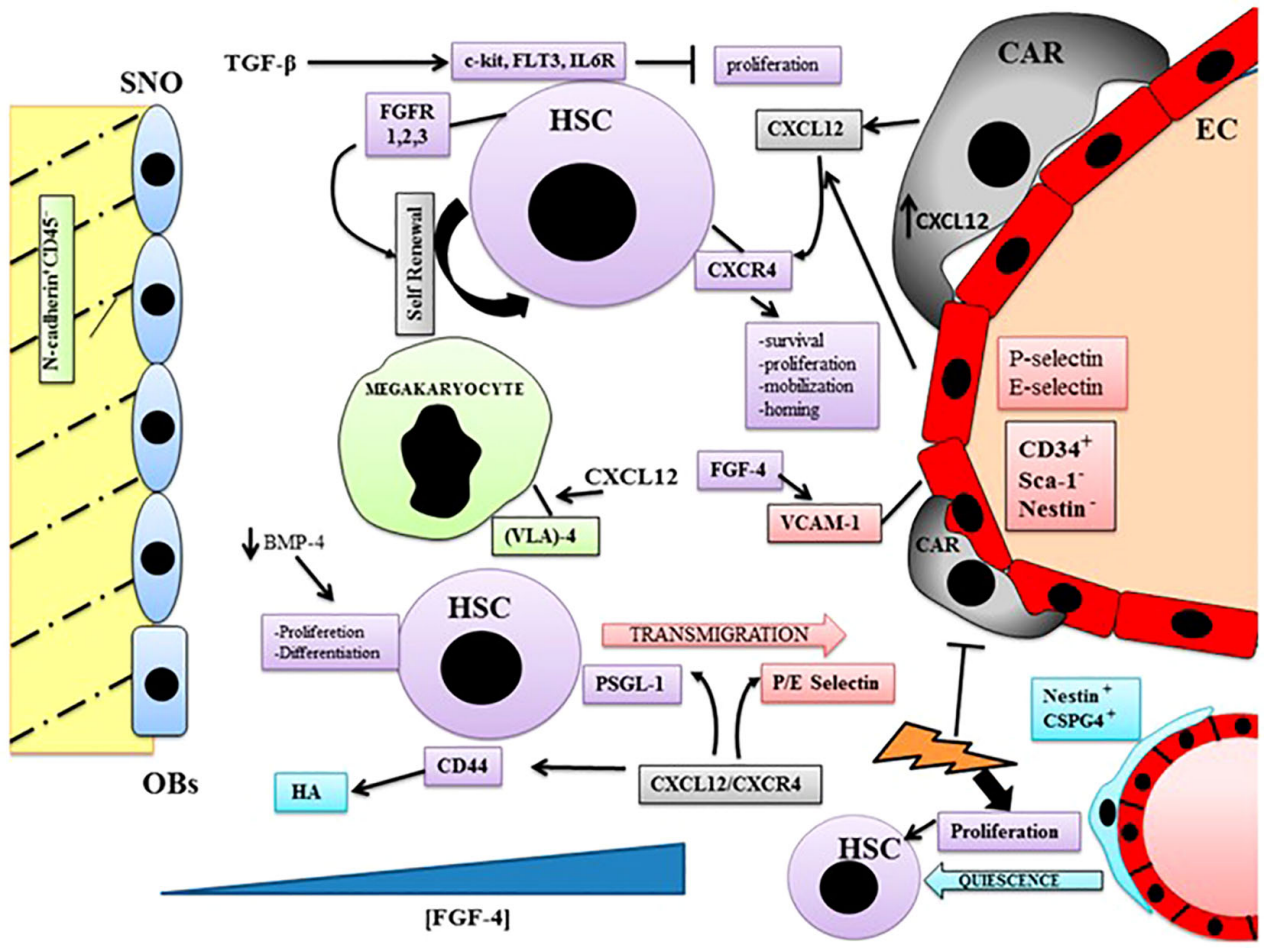
In the vascular niche hematopoiesis occurs in the extravascular spaces between the sinuses. The medullary vascular sinuses are lined with endothelial cells and surrounded by adventitial cells, called CXCL12-abundant reticular (CAR) cells. The closeness between sinusoidal endothelial cells and HSCs is very important for their maturation and so for the hematopoietic process. An arteriolar niche has been found where quiescent HSCs are associated with a cell population different from CAR, named peri-arteriolar nestin cells. These cells express chondroitin sulfate proteoglycan-4 (CSPG4), show chemoresistance after genotoxix injury, and activate HSC proliferation. CXCL12-abundant reticular cells (CAR); fibroblast growth factor receptors (FGFRs); transforming growth factor-β (TGF-β); bone morphogenetic protein (BMP); hyaluronic acid (HA); glycoprotein ligand-1 (PSGL-1); very late antigen (VLA-4); chondroitin sulfate proteoglycan-4 (CSPG4); fibroblast growth factor-4 (FGF-4); P/E selectin type P and E (P/E selectin); vascular cell adhesion molecule 1 (VCAM-1); FMS like tyrosine kinase 3 (FLT3); interleukin 6 receptor (IL6R). Blue triangle: FGF-4 gradient; lightning sign: genotoxic stimulation (Reproduced from 2).

HSCs have the capacity of self-renewal and give rise to all the blood cells and are organized in the endosteal niche, close to the endosteum, and in the vascular niche, close to the vasculature (**Figures 1**, **2**). The endosteum includes osteoblasts, osteoclasts, fibroblasts, macrophages, endothelial cells, and adipocytes (3). In the vascular niche, endothelial cells, pericytes, and smooth muscle cells release molecules involved in the recruitment of HSCs, endothelial precursor cells, and mesenchymal stem cells (4).

During embryogenesis the first hematopoietic cells arise in the extra-embryonic yolk sac. During further development, the production of hematopoietic cells moves to the aorta-gonad-mesonephros region, the placenta, the fetal liver, the spleen, and finally to the bone marrow. After birth the bone marrow becomes the major hematopoietic site. HSCs are sources of instructive signals that maintain and regulate their activity throughout life (5). CD34 is the earliest marker for HSCs to be described. Stem cell factor (SCF) stimulates the proliferation and differentiation of HSCs, and SCF receptor, c-kit (CD 177) is highly expressed on quiescent HSCs in adult bone marrow (6).

The KIT receptor is part of the family of type III receptor tyrosine kinases that include macrophage colony-stimulating factor/receptor (CSF-1R), the platelet-derived growth factor receptor (PDGFR), and the FMS-like tyrosine kinase-3 (FLT-3). These cell-surface receptors have similar structures, including an extracellular immunoglobin-like domain, a transmembrane domain, the juxta membrane region and the cytoplasmic kinase domains.

The bone marrow microenvironment provides the necessary signals for the development from HSCs into mast cell precursors, which then migrate to other sites. The first source of mast cells is the yolk sac. Mast cells derived from the embryonic yolk sac are the first skin and connective tissue mast cells in the embryo (7, 8). Starting in late gestation, embryonic mast cells are gradually replaced by definitive HSC-derived progenitors (9). Mast cells complete their maturation and differentiation in the targeted tissues in the presence of local growth factors including interleukins (IL) -3, -4, -5, -10, -33, CXC motif chemokine 12 (CXCL-12), nerve growth factor (NGF) and transforming growth factor-beta (TGF-β) (10, 11).

SCF and IL-3 are crucial for this process (12–14). After exposure to SCF, HSCs undergo maturation and develop into mature mast cells. Deprivation of SCF results in mast cell growth arrest and apoptosis (15). Administration of human recombinant SCF results in the development of an increased number of mast cells (16).

Mast cells derive from bone marrow precursors, as demonstrated for the first time in 1977–1978 when Kitamura’s group showed reconstitution of mast cells in mast-cell deficient mice by transfer of wild-type bone marrow (17, 18). In these mice, mast cells are absent or reduced because they depend on kit signaling for their growth and survival. By transfer of wild type bone marrow, reconstituted mast cells provide evidence that mast cells are derived from precursors that reside in the bone marrow. Mast cell progenitors express CD34, as other HSCs, and mast cell-related surface markers including c-kit (19).

Development of mast cells in mouse bone marrow occurs along the myeloid pathway. The common myeloid progenitor (CMP) gives rise to either the megakaryocyte-erythrocyte progenitor or to the granulocyte macrophage progenitor (GMP). The GMP gives rise to macrophages, eosinophils, neutrophils or to basophil-mast cell progenitors (BMCPs). BMCPs could be identified as a kit^+^, FC γRII/RIII^+^, β7 integrin^hi^, FcεRI^−^cell that only give rise to mast cells or basophils in culture and transfer of BMCP into mast cell deficient mice led to the appearance of mast cells in the spleen and peritoneal cavity (20).

Only mast cells retain c-kit expression throughout their lifetime, whereas it is lost in other hematopoietic lineages during differentiation. Late mast cell progenitors express IgE receptor (FcεRI) and are less or non-granulated in contrast to mature mast cells which have many methachromatic granules (21).

**Figure 3:**
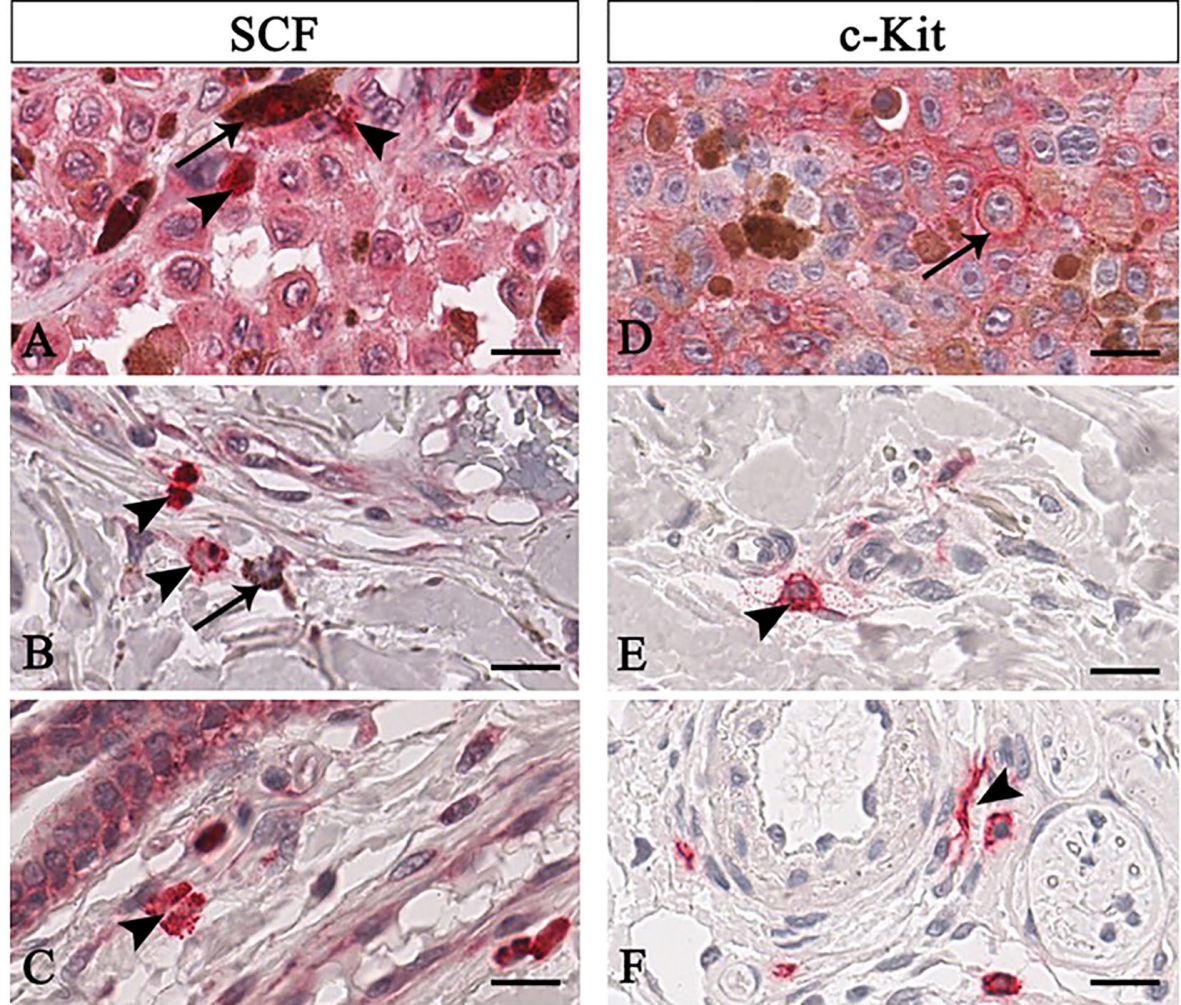
Representative immunohistochemistry of SCF **(A–C)** or c-Kit **(D–F)** in the melanomas tissue sections. Micrographs show pigmented tumor cells (**A**, arrow) or non-pigmented tumor cells but with nucleolated nuclei (**D**, arrow) labelled for both SCF or c-Kit. Instead, SCF^+^/c-Kit^+^ mast cells are rounded-shape and hyperchromatic cells with positive granules in the cytoplasm (**B**, arrowhead) or secreted (**A, C, E** arrowhead). Some mast cells are spindle-shaped, thus resembling fibroblasts (**F**, arrowhead). Scale bar: 60 μm. (Reproduced from 26).

Integrins and cadherins are adhesion molecules that are produced by bone marrow stromal cells (BMSCs). Therefore, in addition to providing SCF, BMSCs participate in the physical anchoring of mast cells within the bone marrow. These molecules promote close intercellular contact and promote mast cell survival and proliferation (22, 23). For better stabilization of mast cells in bone marrow, the β1 integrins promote mast cell interaction with BMSCs and in this way enhance mast cells adhesion to fibronectin in the extracellular matrix. This adhesion also influences mast cell survival, contributing to their increase in the bone marrow (24). BMSCs secrete cytokines and growth factors, including IL-3 and IL-6, and granulocyte-macrophage colony-stimulating factor (GM-CSF) (11, 25), involved in mast cell differentiation and activation and enhancing autocrine and paracrine signaling loops involving the JAK/STAT pathway (26) (**Figure 3**). The excessive proliferation of mast cells can displace HSCs from their niche in bone marrow, which could damage their function and lead to abnormal hematopoiesis (27, 28). IL-6 reduces mast cell expansion and inflammatory signaling within bone marrow (29).

Mast cells interact with fibroblasts and other myeloid cells, such as monocytes, macrophages, and dendritic cells, within the bone marrow. A reciprocal relationship exists between mast cells and fibroblasts through mast cell modulation of fibroblast phenotype mediated by the release of vascular endothelial growth factor (VEGF), IL-4, and fibroblast growth factor-2 (FGF-2), whereas fibroblasts provide signals for mast cell differentiation, survival and activation (30). A crosstalk exists between mast cells and other bone marrow cell types mediated by the release of specific mediators: for monocytes, leukotriene D-4 (LTD-4), VEGF, and platelet activating factor (PAF) (31, 32); for macrophages, IL-6, IL-13, PAF and prostaglandin D-2 (PGD-2) (33, 34); for dendritic cells, PGE-2, PGD-2, VEGFC, IL-13 (31, 35, 36). Macrophages secrete matrix metalloproteinases, which are implicated in extracellular matrix remodeling and facilitate mast cell migration and tissue infiltration (37).

## Mast cells in the bone marrow microenvironment in pathological conditions

The bone marrow microenvironment plays a key role in mast cell diseases, such as systemic mastocytosis, where abnormal mast cell proliferation occurs in the bone marrow, and in other conditions like cancer and bone fracture healing, where mast cells are recruited to the tissue microenvironment. Neoplastic mast cells remain partially responsive to SCF, and increased SCF expression in the microenvironment may further augment aberrant kit signaling and contribute to disease progression (38).

Systemic mastocytosis is a clonal proliferation of mast cells in which there is the involvement of at least one extracutaneous organ with or without skin involvement. Bone marrow is the commonest site involved in systemic mastocytosis. Neoplastic mast cells in the bone marrow are a key feature of systemic mastocytosis. These cells can appear different from normal mast cells, sometimes aggregating, taking on elongated shapes, or forming characteristic “target” lesions. Marrow involvement is seen in both the indolent and aggressive forms of systemic mastocytosis and in systemic mastocytosis with associated clonal hematologic non-mast cell lineage disease, mast cell leukemia and mast cell sarcoma (39). IL-4, produced by mast cells, promotes the differentiation of T cells into the Th2, which may suppress anti-tumor immune responses and contribute to immune evasion in systemic mastocytosis (40).

**Figure 4:**
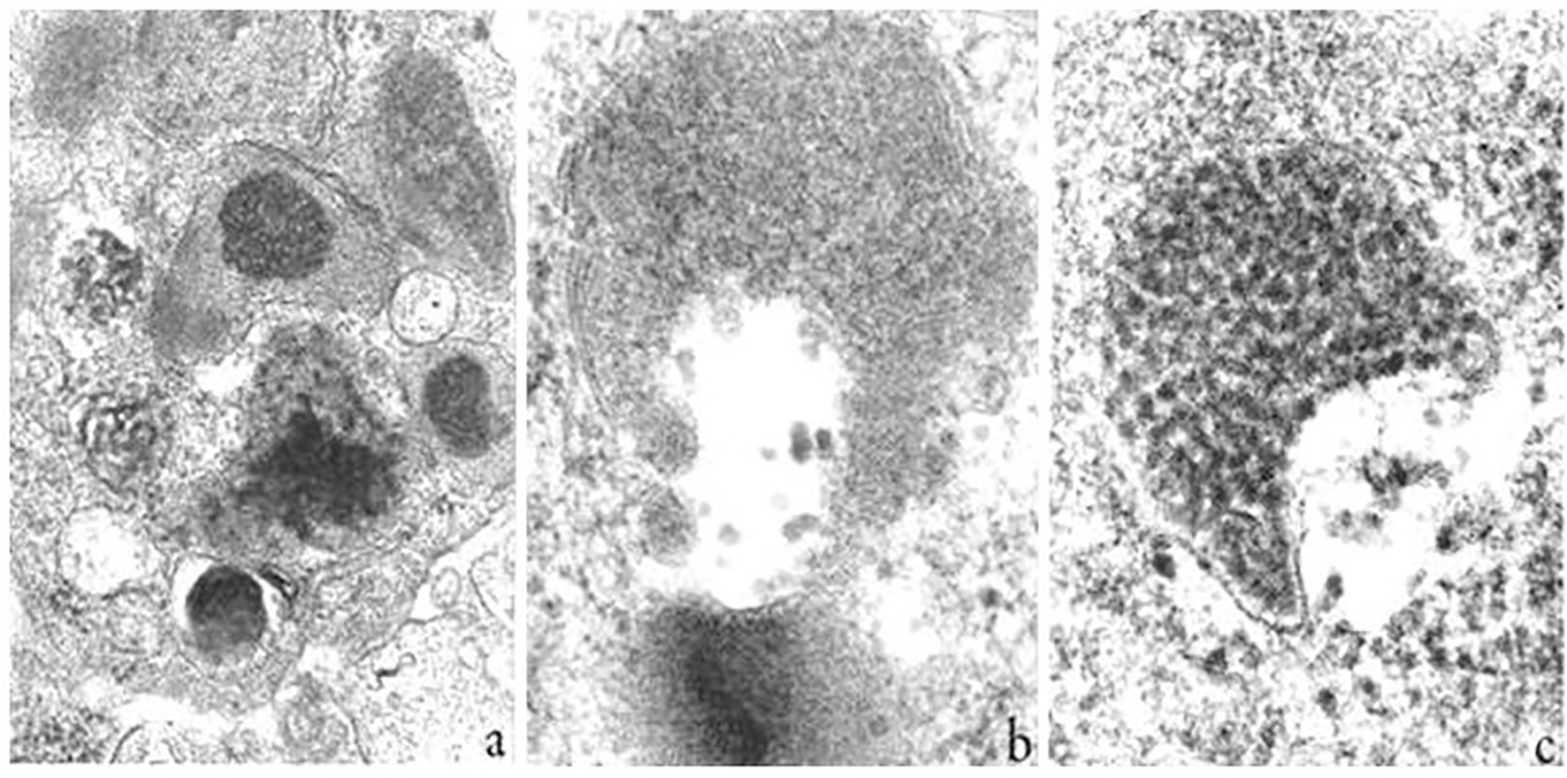
Ultrastructural findings of mast cell granules from high-grade B cell non Hodgkin lymphoma (B-NHL). In **(a)** a heterogeneous mixture consisting of coarse particles and scrolls, thick threads and finely granular material or a mixture of this and scrolls; in **(b)** a semilunar granular aspect and in **(c)** a full complement of dense particles in this type of granule. (Reproduced from 41).

Mast cells are present in most solid tumors, where they may shape the tumor microenvironment and determine therapy response like other immune cells. Increased numbers of mast cells in bone marrow have been described in association with lymphoma (**Figure 4**), hairy cell leukemia, and myeloid neoplasms. In lymphoma, bone marrow mast cells are often increased, correlating with disease severity. They play a role in the tumor microenvironment by promoting tumor growth, angiogenesis, and fibrosis (42). In hairy cell leukemia, increased mast cells in the bone marrow are a reactive phenomenon, not part of the malignant clone itself. They appear alongside the neoplastic cells, along with other changes like reduced hematopoietic elements, dysplastic changes, and fibrosis (43). In chronic myeloid leukemia and Waldenstrom’s macroglobulinemia, mast cells are a prominent feature of the bone marrow microenvironment and can influence disease progression (44).

Neoplastic mast cells may impair hematopoiesis through the secretion of inhibitory cytokines such as tumor necrosis factor-alpha (TNF-α) and TGF-β, which negatively regulate HSCs proliferation and differentiation (45, 46), leading to pancytopenia and disruption across erythroid, myeloid, and lymphoid lineages.

Mast cells promote tumor angiogenesis by expressing pro-angiogenic factors such as VEGF and FGF-2 (47). Bone marrow angiogenesis plays an important role in the pathogenesis and progression of hematological malignancies, and an increased number of mast cells has been demonstrated in angiogenesis associated with multiple myeloma and chronic lymphocytic leukemia (48, 49). Moreover, infiltrating mast cells correlate with angiogenesis in bone metastasis from gastric cancer patients (50).

In several fibrotic diseases, mast cell hyperplasia and degranulation occur. Fibrotic changes in the extracellular matrix affect the biological role of bone marrow, causing changes in mast cells survival and resistance of mast cells to apoptosis (51). The differentiation of extracellular matrix affects migration and localization of mast cells within the bone marrow, promoting their accumulation in specific niches that support their proliferation (52). Targeting TGF-β signaling is a potential strategy to counteract bone marrow fibrosis. Inhibition of TGF-β through inhibition of the SMAD3 pathway, reverses the fibrotic phenotype of bone marrow mast cells (53).

Mast cells are recruited to the site of a bone fracture. Their numbers increase during the initial stages of healing and decline as the bone remodels. Mast cells in the bone marrow are involved in both regulating normal bone fracture healing and negatively impacting it in certain conditions like osteoporosis. They trigger inflammation and regulate the activity of bone-remodeling cells during healing, which is crucial for the process but can lead to complications in diseases with high inflammation. In osteoporotic fractures, mast cells can cause compromised healing by triggering excessive inflammation and stimulating osteoclast activity (54).

## Concluding remarks

Mast cell progenitors originate in the bone marrow from HSC to a GMP, which also gives rise to neutrophils, eosinophils, monocytes, and basophils. Moreover, a bi-potent basophil/mast cell progenitor (BMCP) which can further differentiate *in vitro* to mast cells or basophils, was identified in the spleen. Once released in the bloodstream, mast cells quickly migrate to peripheral tissues where they complete their differentiation.

Mast cells are long-living innate immune cells widely distributed in mucosal and connective tissues. They are located at the interface with the external environment, therefore are among the first cell type that can get in contact with pathogens. It is well known that mast cells are found in every tissue, most abundantly near barriers where they exert the role of immune sentinels during both acute and chronic inflammation. Most mast cells are in the gastrointestinal tract, in the peritoneum, in the skin and in the lungs, and are rarely found in other organs such as lymph nodes, spleen, brain, pancreas, kidneys.

Mast cells are known for their effects when eliciting immediate hypersensitivity reactions. However, they are involved in numerous other physiological and pathological conditions such as those that occur in bone marrow microenvironments, as has been discussed in this article.
